# Synthesis and biological evaluation of 2-(4-methylsulfonyl phenyl) indole derivatives: multi-target compounds with dual antimicrobial and anti-inflammatory activities

**DOI:** 10.1186/s13065-020-00675-5

**Published:** 2020-03-30

**Authors:** Ahmed M. M. Shaker, Eman K. A. Abdelall, Khaled R. A. Abdellatif, Hamdy M. Abdel-Rahman

**Affiliations:** 1 Department of Pharmaceutical Chemistry, Faculty of Pharmacy, Nahda University, Beni-Suef, 62517 Egypt; 2 Department of Pharmaceutical Organic Chemistry, Faculty of Pharmacy, Beni-Suef University, Beni-Suef, 62514 Egypt; 3 Pharmaceutical Sciences Department, IbnSina National College for Medical Studies, Jeddah, 21418 Kingdom of Saudi Arabia; 4 Department of Medicinal Chemistry, Faculty of Pharmacy, Assiut University, Assiut, 71526 Egypt

**Keywords:** Antimicrobial, Indomethacin analogues, COX-2 inhibitors, Nitric oxide, Anti-inflammatory

## Abstract

Three series of 2-(4-methylsulfonylphenyl) indole derivatives have been designed and synthesized. The synthesized compounds were assessed for their antimicrobial, COX inhibitory and anti-inflammatory activities. Compound **7g** was identified to be the most potent antibacterial candidate against strains of *MRSA*, *E. coli, K. pneumoniae, P. aeruginosa,* and *A. baumannii*, respectively, with safe therapeutic dose. Compounds **7a–k, 8a–c,** and **9a–c** showed good anti-inflammatory activity with excessive selectivity towards COX-2 in comparison with reference drugs indomethacin and celecoxib. Compounds **9a–c** were found to release moderate amounts of NO to decrease the side effects associated with selective COX-2 inhibitors. A molecular modeling study for compounds **7b, 7h,** and **7i** into COX-2 active site was correlated with the results of in vitro COX-2 inhibition assays.

## Introduction

Bacterial resistance reached a dangerous level due to the misuse of antibiotics thus searching for new antimicrobial agents is a significant issue [1]. Furthermore, the administration of multiple drugs to relieve inflammation associated with a bacterial infection may have some secondary health problems and may increase adverse effects [2]. Unfortunately, few drugs possessed these two activities in a single compound. Therefore, there are continuous trails to develop a monotherapy against inflammation due to microbial infection (dual antimicrobial/anti-inflammatory agent) with minimal adverse effects and high safety margin [3].

**Fig. 1:**
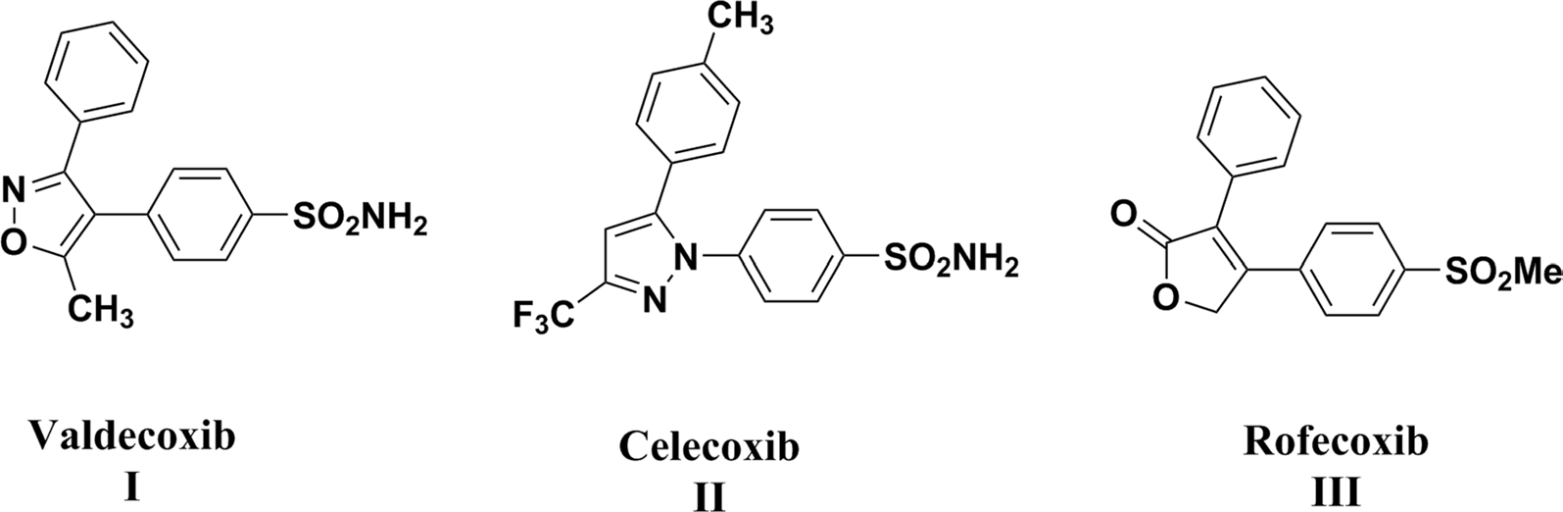
Chemical structures of selective cyclooxygenase-2 (COX-2) inhibitor drugs (**I, II, III**)

The nonsteroidal anti-inflammatory drugs (NSAIDs) are used as the primary remedy for pain, fever, and inflammation through inhibition of cyclooxygenase (COX) enzymes [4–6]. Selective COX-2 inhibitor drugs like valdecoxib **I**, celecoxib **II** and rofecoxib **III** relieve inflammation without any gastric side effects [7] (Fig. 1). Despite less gastric irritation of selective COX-2 inhibitors, they showed a few cardiovascular issues consisting of myocardial infarction and high blood pressure [8, 9], leading to the withdrawal of both rofecoxib and valdecoxib from the market [10]. The cause of cardiovascular issues may be due to inhibition of vasodilatory prostacyclin (PGI_2_) and an increase in the level of platelet activator thromboxane A_2_ (TxA_2_) [11]. Nitric oxide (NO) showed vasodilator activity and inhibition of platelet aggregation [12]. Accordingly, attachment of NO donor moiety to selective COX-2 inhibitors may be beneficial to overcome the cardiovascular side effects [13, 14].

A lot of biologically aryl hydrazone derivatives with antimicrobial activity are found in many literatures [15–17] which include nitrofurantoin **IV** [18, 19]. Additionally, indole-based indomethacin **V** is a potent NSAID used for the treatment of inflammatory diseases such as rheumatoid arthritis and osteoarthritis [20]. Still, due to its high selectivity for COX-1 inhibition and its acidic nature, it had an apparent ulcerogenic effect [21].

**Fig. 2:**
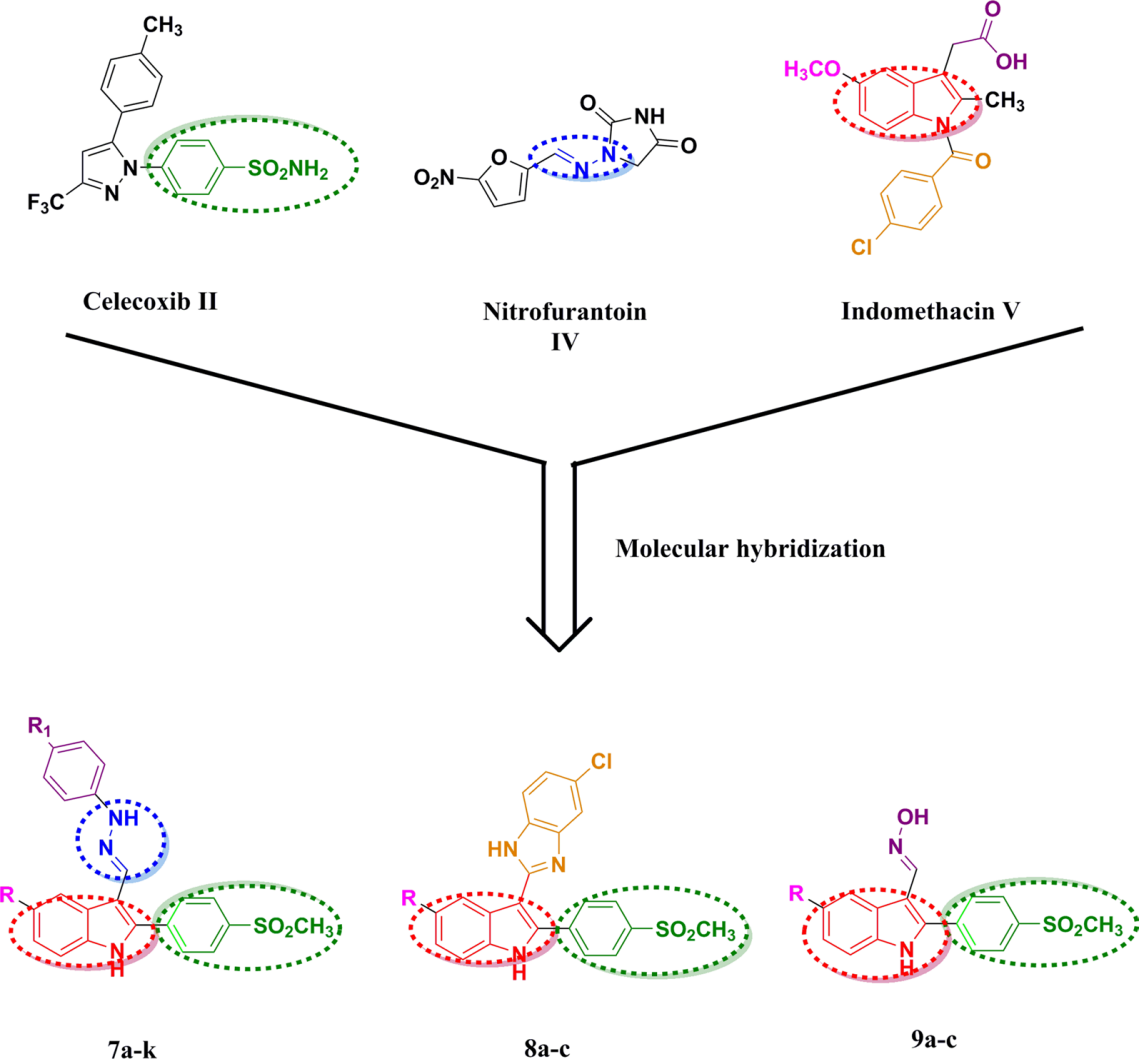
Hybridization of chemical structures of indomethacin **V,** celecoxib **II,** nitrofurantoin **IV** to design indole derivatives **7a–k, 8a–c, and 9a–c**

Herein, we aimed to make molecular hybridization of the indole part of indomethacin with *p*-methylsulfonyl phenyl part of selective COX-2 inhibitors to match the overall structure of coxibs [presence of a diaryl heterocycle bearing one sulfonamide (SO_2_NH_2_) or methylsulfonyl (SO_2_CH_3_) group] [22]. Keep in mind the presence of arylhydrazone derivatives at position 3 in indole with the hope to get compounds with dual antimicrobial/anti-inflammatory activity (Fig. 2).

## Results and discussion

### Chemistry

**Scheme 1:**
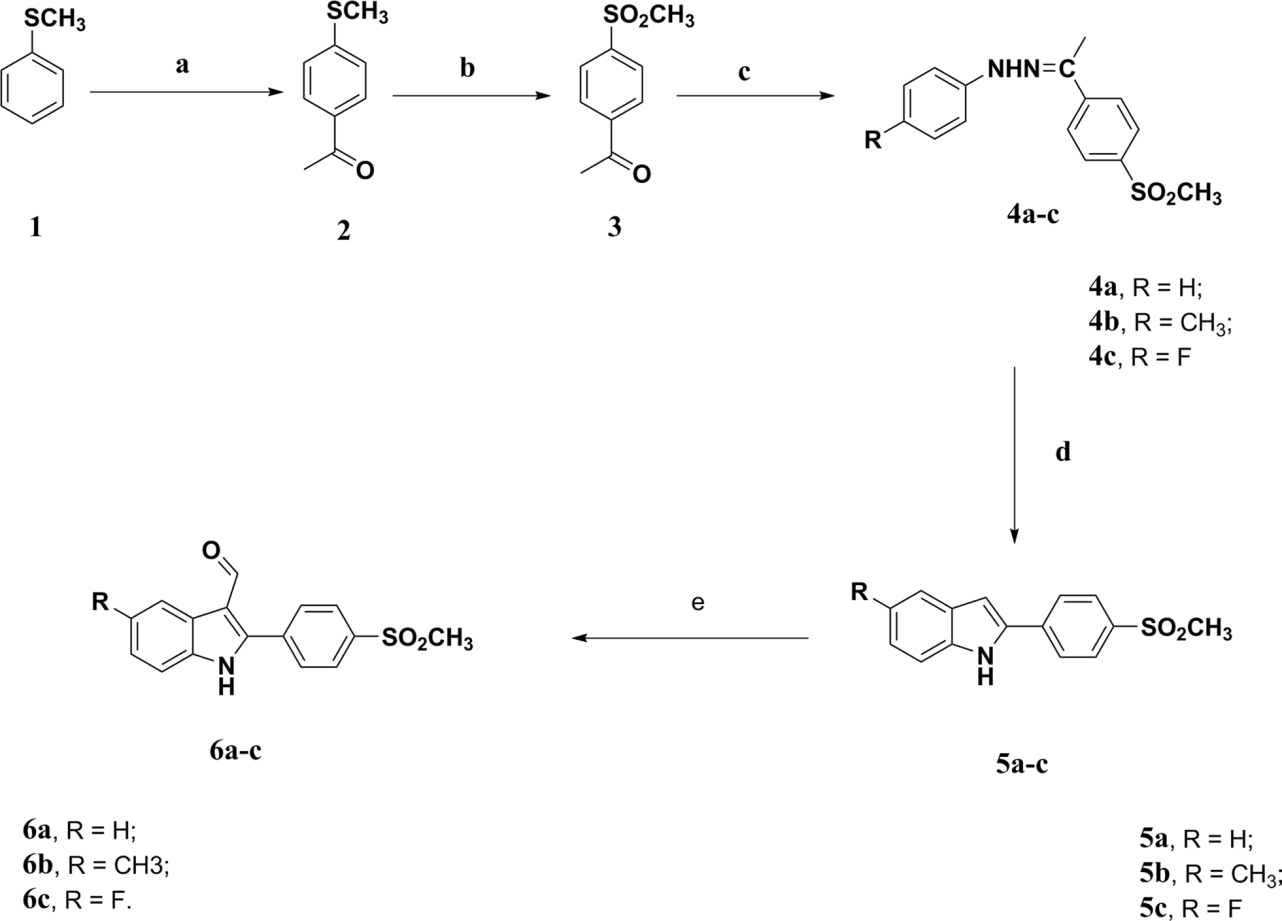
Reagents and conditions: **a** acetic anhydride, AlCl_3_; **b** Oxone, H_2_O, reflux, 18 h; **c** 4-substituted phenylhydrazine HCl, ethanol, reflux, 4 h; **d** PPA, water bath, 4 h; **e** POCl_3_, DMF, RT, overnight

The compounds were synthesized through a series of reactions illustrated in Scheme 1, 2. The reaction of *p*-methylsulfonyl acetophenone (**3**) with 4-un/substituted phenylhydrazine HCl under Fischer indole synthesis conditions yielded indole derivatives (**5a–c**) that are converted to indole-3-carbaldehyde derivatives (**6a–c**) by Vilsmeir Haack’s formylation reaction using POCl_3_ and DMF (Scheme 1).

**Scheme 2:**
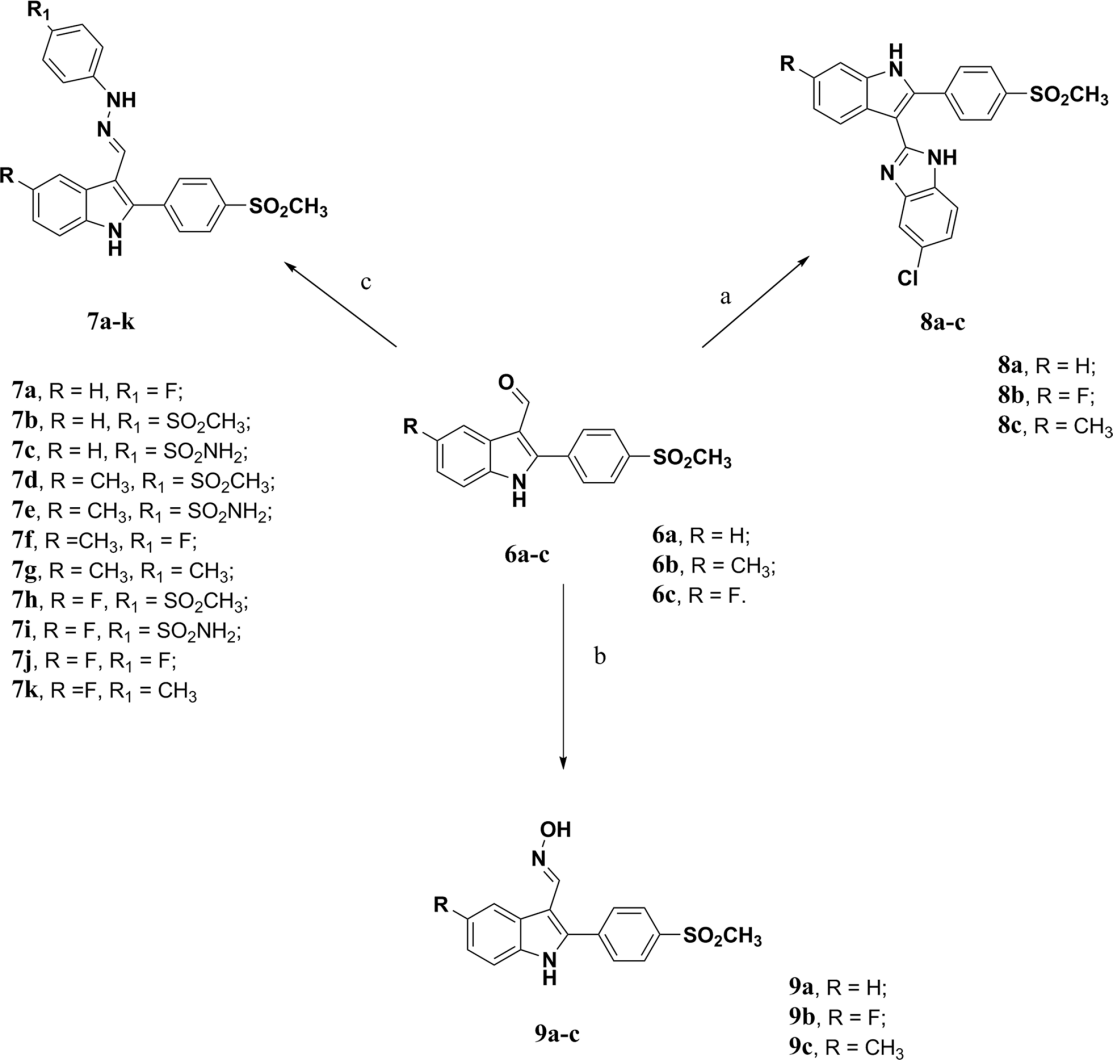
Reagents and conditions: **a** 4-chloro-*o*-phenylenediamine, Na_2_S_2_O_5_, DMF, reflux, 6 h; **b** 4-substituted phenylhydrazine HCl, ethanol, reflux, 4–6 h.; **c** hydroxylamine, ethanol, few drops of pyridine, reflux, 4–6 h

IR spectra for compounds **6a–c** showed significant bands at 3205–3320 cm^−1^ of indole NH, 1657–1670 cm^−1^ of C=O and 1150, 1300 cm^−1^ of SO_2_. ^1^H NMR spectra showed a signal at δ 10.00–10.04 ppm of an aldehydic proton (H-C=O), 3.17–3.21 ppm of SO_2_CH_3_ and 12.92–12.62 ppm of indole NH which is D_2_O exchangable.

Indole-3-carbaldehyde derivatives (**6a–c**) were reacted with 4-substituted phenylhydrazine HCl to give hydrazone derivatives (**7a–k**) in good yield. The structure elucidation of hydrazone derivatives (**7a–k**) was based on IR, ^1^H NMR, and ^13^C NMR spectral data. IR spectra showed bands at 1593-1597 cm^−1^ for C=N and disappearance of the carbonyl absorption band at 1657–1670 cm^−1^ which confirm hydrazone formation. ^1^H NMR spectra showed a signal at δ 8.24–8.36 ppm of hydrazone proton (H-C=N), 10.03–10.73 ppm of hydrazone NH which is D_2_O exchangeable, 12.00 ppm for NH indole which is D_2_O exchangeable and disappearance of an aldehydic proton at δ 10.00–10.04 ppm which confirm hydrazone formation. ^13^C NMR spectra showed a peak at 143–149 ppm of hydrazone carbon (C=N) which confirm hydrazone formation.

On the other hand, benzimidazole derivatives (**8a–c)** are synthesized from the reaction of Indole-3-carbaldehyde derivatives (**6a–c**) with 4-chloro-*o*-phenylenediamine in the presence of sodium metabisulphite. IR spectra showed bands at 3272–3382 cm^−1^ (indole NH, benzimidazole NH) and disappearance of the carbonyl absorption band at 1657–1670 cm^−1^. ^1^H NMR spectra showed the disappearance of an aldehydic proton at δ 10.00–10.04 ppm and the appearance of a signal at δ (12.37–12.45) ppm of benzimidazole NH (D_2_O exchangeable) in addition to a signal at δ 12.04–12.18 ppm of indole NH (D_2_O exchangeable).

Oxime derivatives (**9a–c)** resulted from the reflux of the reaction of Indole-3-carbaldehyde derivatives (**6a–c**) with hydroxylamine HCl. IR spectra lacked the carbonyl absorption band at 1657–1670 cm^−1^ and showed absorption bands at 3272–3382 cm^−1^ (NH, OH) and 1597 cm^−1^ (C=N). ^1^H NMR spectra showed a singlet signal at δ 8.32 ppm of azomethine proton H-C=N, 10.89 ppm of OH (D_2_O exchangeable) in besides to signal at δ 11.79–12.04 ppm of indole NH (D_2_O exchangeable) and disappearance of an aldehydic proton at δ 10.00–10.04 ppm which confirm oxime formation.

### Biological evaluation

#### Antimicrobial screening

The antimicrobial study was performed by CO-ADD (The Community for Antimicrobial Drug Discovery), funded by the Wellcome Trust (UK) and The University of Queensland (Australia). Evaluation of all synthesized compounds for their antimicrobial activities was done against five pathogenic bacteria, *methicillin*-*resistant Staphylococcus aureus* (ATCC 43300) as Gram-positive bacteria, *Escherichia coli* (ATCC 25922), *Klebsiella pneumonia* (ATCC 700603), *Acinetobacter baumannii* (ATCC 19606) and *Pseudomonas aeruginosa* (ATCC 27853) as Gram-negative bacteria and antifungal activity against two pathogenic fungal strains *Candida albicans* (ATCC 90028) and *Cryptococcus neoformans var. grubii* (H99; ATCC 208821) (Table 1).

**Table 1:** The antibacterial and antifungal activities (growth inhibition %) for compounds **7a–k**, **8a–c** and **9a–c** at 32 µg/mL concentration

Compound No.	**Sa**^**a**^	**Ec**^**b**^	**Kp**^**c**^	**Pa**^**d**^	**Ab**^**e**^	**Ca**^**f**^	**Cn**^**g**^
**7a**	95.76	96.48	97.64	97.76	96.66	6.28	− 64.35
**7b**	25.62	− 8.09	− 5.34	3.8	35.54	9.55	− 280.7
**7c**	21.88	3.17	12.8	11.3	43.29	4.13	− 110.9
**7d**	15.6	− 5.77	4.86	− 8.11	34.95	2.54	− 177.2
**7e**	7.58	− 11.49	− 14.6	− 22.55	43.64	28.66	− 59.93
**7f**	8.33	− 9.43	8.05	− 7.9	66.69	3.5	− 118.8
**7g**	96.15	86.42	87.53	94.63	85.76	4.88	− 57.42
**7h**	30.26	− 13.86	23.59	7.97	51.82	25.34	− 99
**7i**	95.22	96.45	94.4	96.93	94.34	15.32	− 104.5
**7j**	30.59	− 2.24	12.27	− 0.85	46.23	1.91	− 80.19
**7k**	28.25	− 0.72	8.77	2.23	31.42	1.79	− 55.44
**8a**	13.6	− 45.65	− 22.34	− 28.34	− 15.51	7.71	− 292.1
**8b**	11.28	− 8.78	6.56	14.46	22.42	13.22	− 114.4
**8c**	4.62	− 25.19	− 8.38	− 10	− 13.94	1.65	− 288.1
**9a**	− 3.37	− 12.05	14.84	2.49	42.1	9.15	− 254
**9b**	10.33	− 5.88	9.23	7.9	33.26	2.47	− 119.3
**9c**	− 2.72	− 15.45	1.19	− 0.83	33.66	4.88	− 136.1

^a^*MRSA*

^b^*E. coli*

^c^*K. pneumoniae*

^d^*P. aeruginosa*

^e^*A. baumannii*

^f^*C. albicans*

^g^*C. neoformans var. grubii*

Results revealed that hydrazone derivatives **7c, 7e, 7f, 7** **h,** and **7j** have moderate antibacterial activity against Gram-negative *A. baumannii* with growth inhibition 43.29, 43.64, 66.69, 51.82 and 46.23%, respectively. While the hydrazone derivatives **7a, 7g,** and **7i** have high antibacterial activity against *MRSA* bacteria and *E. coli, K. pneumoniae, P. aeruginosa,* and *A. baumannii* with growth inhibition ranged from 85.76 to 97.76%.

Additionally, the oxime derivatives **9a** showed moderate antibacterial activity against Gram-negative *A. baumannii* with growth inhibition 42.1%, while benzimidazole derivatives (**8a–c**) showed weak antibacterial activity.

On the other hand, all compounds have weak antifungal activity against *C. albicans* and *C. neoformans var. grubii.*

Minimal inhibitory concentrations (MIC µg/mL) measurements were performed for compounds with significant microbial growth inhibition (**7a, 7g,** and **7i)** using ceftriaxone and amphotericin B as a reference drug for antibacterial and antifungal activity, respectively.

As shown in Table 2, compounds **7a, 7g** and **7i** have the best antibacterial activity comparable to that of ceftriaxone against *MRSA*, *E. coli, K. pneumoniae, P. aeruginosa* and *A. baumannii*, respectively.

**Table 2:** Minimum inhibitory concentrations (MIC µg/mL) of most active compounds **7a, 7g, 7i** and reference drugs, ceftriaxone and amphotericin B

Compound No.	**Sa**^**a**^	**Ec**^**b**^	**Kp**^**c**^	**Pa**^**d**^	**Ab**^**e**^	**Ca**^**f**^	**Cn**^**g**^	**CC**_**50**_^**h**^	**HC**_**10**_^**i**^
**7a**	8	≤ 0.25	8	4	16	> 32	> 32	> 32	> 32
**7g**	1	≤ 0.25	1	1	4	> 32	> 32	4.2	> 32
**7i**	2	≤ 0.25	2	2	4	> 32	> 32	2.987	> 32
**Ceftriaxone**	32	0.125	16	32	32	NT^j^	NT	NT	NT
**Amphotericin B**	NT	NT	NT	NT	NT	1.56	1.56	NT	NT

^a^*MRSA*

^b^*E. coli*

^c^*K. pneumoniae*

^d^*P. aeruginosa*

^e^*A. baumannii*

^f^*C. albicans*

^g^*C. neoformans var. grubii*

^h^CC_50_ is the concentration at 50% cytotoxicity

^i^HC_10_ is the concentration at 10% hemolysis

^j^Not tested

The safety margin for the active compounds to human cells was determined through cytotoxicity against human embryonic kidney cell line and hemolysis of human red blood cells. The tested compounds **7a, 7g,** and **7i** were tolerated and non-toxic to human cells as the cytotoxic and hemolytic dose was higher than the therapeutic dose (Table 2).

Compound **7a** lacked general nonspecific toxicity, as the largest therapeutic dose (16 µg/mL against *A. baumannii*) was lower than the cytotoxic and hemolytic concentration (> 32, > 32 µg/mL respectively). Also, compound **7g** showed safe therapeutic concentration against all tested microbes except for *A. baumannii* (4 µg/mL) which is near to cytotoxic concentration (4.2 µg/mL). Otherwise, the therapeutic concentration of compound **7i** against all tested microbes was safe except for *A. baumannii* (4 µg/mL), which is higher than the cytotoxic concentration (2.987 µg/mL).

#### In vitro cyclooxygenase (COX) inhibition assay

The in vitro assay evaluated the ability of compounds **7a–k, 8a–c,** and **9a–c** to inhibit Ovine COX-1 and human recombinant COX-2. All tested compounds have weak COX-1 inhibition activity (IC_50_ = 9.14–13.2 µM) in comparison with indomethacin (IC_50_ = 0.039 µM). They also exerted potent COX-2 inhibitory activity (IC_50_ = 0.1–0.31 µM) with high COX-2 selectivity (SI = 132–31.29) in comparison with reference drugs, indomethacin and celecoxib.

Hydrazone derivatives **7a–k** showed potent COX-2 inhibitory activity (IC_50_ = 0.10–0.31 µM) with high selectivity (SI = 132–31.29) more than other compounds. Likewise, benzimidazole **8a–c** and oxime derivatives **9a–c** showed good COX-2 inhibitory activity (IC_50_ = 0.13–0.35 µM) in comparison with reference drugs.

Generally, all tested compounds were more selective toward the COX-2 enzyme (SI = 31.29–132) than indomethacin (SI = 0.079) (Table 3) because the size of synthesized compounds was too large to fit into the small COX-1 active site in addition to the presence of diaryl structure bearing SO_2_CH_3_ or SO_2_NH_2_ group.

**Table 3:** In vitro COX-1 and COX-2 inhibition for compounds **7a–k**, **8a–c, 9a–c** and reference drugs

Compounds	COX Inhibition (IC_50_ µM)		Selectivity index^a^ (SI)
	COX-1	COX-2	
**Celecoxib**	14.80	0.05	296
**Indomethacin**	0.039	0.49	0.079
**7a**	10.32	0.11	93.81
**7b**	12.41	0.10	124.10
**7c**	11.41	0.11	103.72
**7d**	10.40	0.15	69.33
**7e**	9.70	0.31	31.29
**7f**	9.73	0.17	57.23
**7g**	7.90	0.20	39.50
**7h**	12.40	0.11	112.72
**7i**	13.20	0.10	132
**7j**	10.80	0.11	98.18
**7k**	8.24	0.21	39.20
**8a**	10.64	0.13	81.84
**8b**	9.41	0.15	62.73
**8c**	11.23	0.12	93.58
**9a**	10.64	0.13	81.84
**9b**	9.42	0.21	44.85
**9c**	8.24	0.24	34.33

^a^Selectivity index (COX-1 IC_50_/COX-2 IC_50_)

#### In vivo anti-inflammatory activity

The results listed in (Table 4) showed that compounds **7a–k, 8a–c,** and **9a–c** offered good anti-inflammatory activity (56.4–93.5% reduction of inflammation) after 6 h in comparison with celecoxib and indomethacin (94.7, 96.6% reduction of inflammation, respectively) after 6 h.

**Table 4:** Anti-inflammatory activities for compounds **7a–k**, **8a–c, 9a–c** and reference drug in carrageen-induced rat paw edema test

Compound	(Edema inhibition %) Edema thickness (mm) ± SEM^a^			
	1 h	3 h	6 h	
**Control**	2.624 ± 0.255	2.232 ± 0.235	1.875 ± 0.181	
**Indomethacin**	70.7	96	96.6	
	0.768 ± 0.050	0.075 ± 0.007	0.075 ± 0.004	
**Celecoxib**	68.9	95.5	94.7	
	0.810 ± 0.074	0.100 ± 0.009	0.100 ± 0.005	
**7a**	74.1	88.7	92	
	0.679 ± 0.03	0.250 ± 0.021	0.151 ± 0.007	
**7b**	76.1	91.2	93.5	
	0.627 ± 0.045	0.196 ± 0.017	0.123 ± 0.009	
**7c**	62.7	81.2	82.5	
	0.978 ± 0.071	0.419 ± 0.028	0.331 ± 0.011	
**7d**	51.6	77.5	78.6	
	1.270 ± 0.015	0.502 ± 0.044	0.405 ± 0.018	
**7e**	53.9	71.1	79.9	
	1.209 ± 0.11	0.645 ± 0.01	0.381 ± 0.007	
**7f**	60.5	72.7	79.2	
	1.036 ± 0.009	0.609 ± 0.03	0.394 ± 0.01	
**7g**	58.5	72.3	67.4	
	1.088 ± 0.090	0.618 ± 0.010	0.618 ± 0.045	
**7h**	75.4	89.4	92.7	
	0.645 ± 0.058	0.236 ± 0.008	0.138 ± 0.006	
**7i**	73.1	94.3	90.1	
	0.705 ± 0.047	0.127 ± 0.009	0.187 ± 0.015	
**7j**	64.3	78.6	71	
	0.936 ± 0.064	0.477 ± 0.027	0.549 ± 0.013	
**7k**	55.7	69.1	66.3	
	1.162 ± 0.088	0.689 ± 0.011	0.638 ± 0.051	
**8a**	52.5	58	56.4	
	1.246 ± 0.076	0.937 ± 0.046	0.826 ± 0.078	
**8b**	52.1	68.1	76.2	
	1.256 ± 0.074	0.712 ± 0.066	0.451 ± 0.013	
**8c**	67.9	71.9	74.2	
	0.842 ± 0.062	0.627 ± 0.05	0.489 ± 0.032	
**9a**	77.2	61.8	62.3	
	0.598 ± 0.050	0.852 ± 0.073	0.714 ± 0.052	
**9b**	66.2	80.5	73.4	
	1.175 ± 0.057	0.700 ± 0.024	0.726 ± 0.055	
**9c**	55.2	68.6	61.7	
	0.886 ± 0.077	0.435 ± 0.033	0.504 ± 0.009	

^a^Each value represents mean ± SEM (n = 4)

Hydrazone derivatives **(7a–k)** showed good anti-inflammatory activity (66.3–93.5% reduction of inflammation) after 6 h, Compounds that contained two SO_2_CH_3_ groups or one SO_2_CH_3_ and one SO_2_NH_2_ group (**7b, 7c, 7d, 7e, 7h,** and **7i**) showed a reduction of inflammation by 93.5, 82.5, 78.6, 79.9, 92.7 and 90.1% after 6 h, respectively, more than other derivatives.

Also, benzimidazole and oxime derivatives **(8a–c, 9a–c)** showed good inhibition of inflammation ranged from 56.4 to 76.2% after 6 h.

Compounds **7b, 7c, 7h** and **7i** that showed the highest COX-2 inhibitory activity (IC_50_ = 0.1, 0.11, 0.11 and 0.1 respectively) with high selectivity (S.I. = 124.2, 103.7, 112.7 and 132 respectively) were found to have excellent anti-inflammatory activity (edema inhibition = 93.5, 82.5, 92.7 and 90.1%, respectively) after 6 h.

#### In vitro nitric oxide release

The NO-releasing properties of compounds **9a–c** were assessed in phosphate buffer of pH 7.4 with Griess reagent [23]. As shown in Table 5, compounds **9a–c** were found to release moderate amounts of NO compared to the sodium nitrite standard solution, which may explain that the desired action of NO is mediated systemically in the biological system [24]. Therefore, the insertion of nitric oxide releasing group (oxime) can offer a method to decrease the cardiovascular side effects of selective COX-2 inhibitors.

**Table 5:** The amount of NO released from tested compounds **9a–c** in phosphate buffer pH = 7.4 (% mol/mol)

Compound No.	Amount of NO released (% mol/mol) ± standardization error (in phosphate buffer PH 7.4)				
	1 h	2 h	3 h	4 h	5 h
**9a**	0.027 ± 0.002	0.065 ± 0.002	0.194 ± 0.007	0.165 ± 0.002	0.138 ± 0.004
**9b**	0.086 ± 0.001	0.147 ± 0.003	0.210 ± 0.002	0.198 ± 0.003	0.218 ± 0.005
**9c**	0.061 ± 0.001	0.130 ± 0.002	0.187 ± 0.001	0.198 ± 0.003	0.225 ± 0.002

#### Structure–activity relationship

Presence of arylhydrazone moiety **7a–k** at position 3 of indole can possess antimicrobial activity against strains of Gram-positive *MRSA* bacteria and Gram-negative *E. coli, K. pneumoniae, P. aeruginosa,* and *A. baumannii* beside their COX-2 inhibitory activity.

Concerning the anti-inflammatory activity, replacement of methyl group in position 2 in indomethacin by *p*-methylsulfonyl phenyl moiety increased COX-2 selectivity through increasing the interaction with a hydrophobic residue of COX-2 active site [25]. In addition, the presence of two SO_2_CH_3_ groups or one SO_2_CH_3_ and one SO_2_NH_2_ group (**7b, 7c, 7d, 7e, 7h,** and **7i**) has COX-2 selectivity more than other derivatives.

Replacement of acidic center (CH_2_COOH) moiety in position 3 in indomethacin by benzimidazole moiety **8a–c,** as a rigid isostere of *p*-chlorobenzoyl moiety of indomethacin, enhances the anti-inflammatory activity and COX-2 selectivity.

### Molecular modeling

To understand the nature of the interaction of the most active synthesized compounds and COX-2 active site, a molecular docking study was performed using crystal structure data for COX-2 (PDB: ID 3LN1) active site obtained from protein data bank [26]. Molecular modeling of compounds **7h, 7i, 7b,** and co-crystallized ligand, celecoxib was performed using MOE 2018.0101 modeling software.

**Fig. 3:**
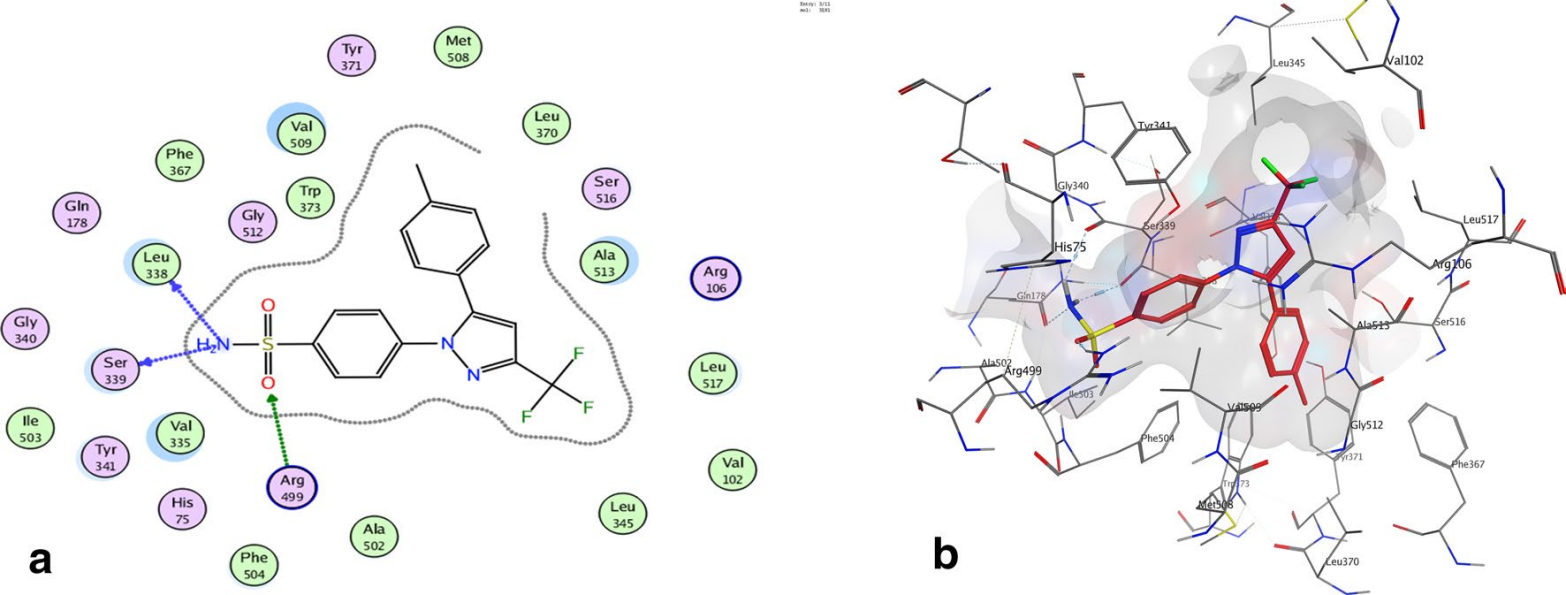
Binding of celecoxib inside COX-2 active site. **a** 2D interaction, the most important amino acids are shown together with their respective numbers. **b** The 3D proposed binding mode inside the active site of COX-2 resulted from docking

**Fig. 4:**
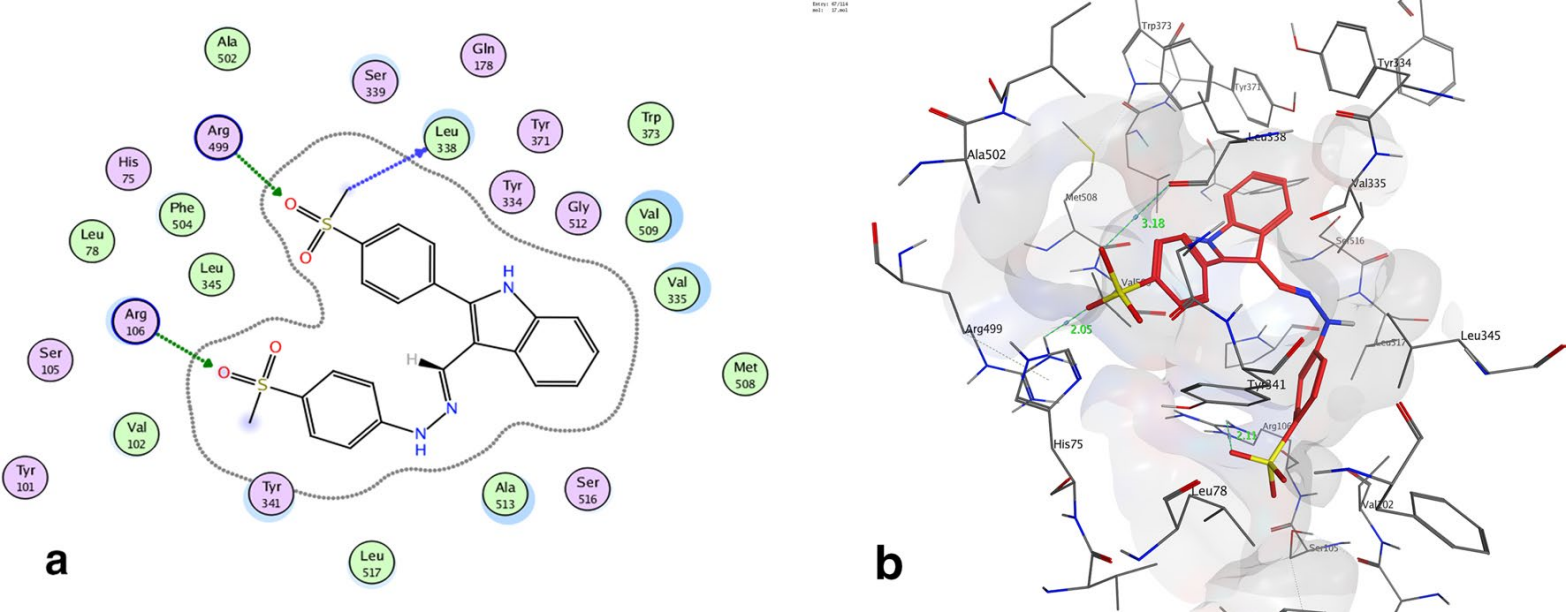
Binding of compound **7b** inside COX-2 active site. **a** 2D interaction, the most important amino acids are shown together with their respective numbers. **b** The 3D proposed binding mode inside the active site of COX-2 resulted from docking

The docking results of compounds **7h, 7i, 7b,** and celecoxib were presented in (Table 6). Hydrazone derivatives **7b, 7h,** and **7i** have been fully fitted within COX-2 active site with high affinity (− 17.19, − 16.71 and − 16.42 kcal/mol, respectively) in assessment with celecoxib (− 14.12 kcal/mol). Compounds **7b, 7h,** and **7i** contained one SO_2_CH_3_ and one SO_2_NH_2_ group or two SO_2_CH_3_ groups that formed hydrogen bonds with different amino acids (Leu338, Arg499, Ser339, Val335, Arg106, and His75). Besides, the indole ring of compound **7h** and **7i** offered hydrophobic interaction with Val509 (Fig. 3, 4). Thus, the molecular docking results ensure that compounds **7b, 7h** and **7i** bind to COX-2 active site with the same manner of celecoxib.

**Table 6:** Molecular docking data for compounds **7b**, **7h**, **7i** and celecoxib in COX-2 active site (PDB ID: 3LN1)

Compound No.	COX-2					
	Affinity (kcal/mol)	Affinity kcal/mol	Distance (in A^o^) from main residue		Functional group	Interaction
**Celecoxib**	− 14.12	− 2.7	3.07	Leu338	–NH_2_	H-donor
		− 1.6	2.99	Ser339	–NH_2_	H-donor
		− 0.8	3.54	Arg499	–SO_2_	H-acceptor
**7b**	− 17.198	− 1.5	3.18	Leu338	–SO_2_CH_3_	H-donor
		− 0.7	2.70	Arg499	–SO_2_	H-acceptor
		− 2.3	2.84	Arg106	–SO_2_	H-acceptor
**7h**	− 16.71	− 1.4	3.23	Leu338	–SO_2_CH_3_	H-donor
		− 2.7	2.83	Arg499	–SO_2_	H-acceptor
		− 0.6	4.71	Val509	–Ph-ring	H-pi
**7i**	− 16.42	− 0.9	3.36	Val335	–NH	H-donor
		− 0.6	3.47	Ser339	–SO_2_CH_3_	H-donor
		− 4.5	2.86	His75	–SO_2_	H-acceptor
		− 1.5	2.94	Arg106	–SO_2_	H-acceptor
		− 0.9	3.77	Val509	–Ph-ring	H-pi

## Conclusion

Three series of 2-(4-methylsulfonylphenyl) indole derivatives **7a–k, 8a–c,** and **9a–c** were evaluated for their antimicrobial and anti-inflammatory activities.

The results showed that arylhydrazone derivatives **7a–k** exhibited moderate to good levels of antimicrobial activity. In particular, compounds **7a, 7g,** and **7i** showed the highest antimicrobial activity against strains of *MRSA* bacteria and many species of Gram-negative with growth inhibition ranged from 85.76 to 97.76%.

Regarding anti-inflammatory activity, all synthesized compounds **7a–k, 8a–c** and **9a–c** showed potent anti-inflammatory (56.4–93.5% reduction of inflammation after 6 h.) and selective COX-2 inhibitory activity (IC_50_ = 0.1–0.31 µM, SI = 132–31.29) more than indomethacin. Besides, oxime derivatives **9a–c** showed good selective COX-2 inhibitory activity with moderate in vitro nitric oxide release, which can offer valuable drug design to decrease the cardiovascular problems.

The molecular modeling study ensured in vitro COX-2 inhibition assay results. Compounds **7b, 7h,** and **7i** fitted to a COX-2 enzyme similar to celecoxib.

These results suggested that the presence of methylsulfonyl moiety in the indole ring offered an increase in COX-2 selectivity more than the reference drug indomethacin. Also, hybridization of methylsulfonyl and arylhydrazone moiety with an indole ring, providing valuable design for the development of compounds with dual antimicrobial/anti-inflammatory activity. Many investigations are currently undergoing to determine the mechanism of action of these compounds.

## Experimental

### Chemistry

A Thomas-Hoover capillary apparatus used to determine melting points. Infrared (IR) spectra were recorded as films on KBr plates using the FT-IR spectrometer.

Thin-layer chromatography (Merck, Darmstadt, Germany) was used for monitoring the reaction mixture, purity, and homogeneity of the synthesized compounds. UV was used as the visualizing agent.

^1^H NMR and ^13^C NMR spectra were measured on a Bruker Avance III 400 MHz for ^1^H NMR and 100 MHz for ^13^C NMR (Bruker AG, Switzerland) with BBFO Smart Probe and Bruker 400 AEON Nitrogen-Free Magnet, Faculty of Pharmacy, Beni-Suef University, Egypt in DMSO-*d*_*6*_ with TMS as the internal standard, where *J* (coupling constant) values are estimated in Hertz (Hz) and chemical shifts were recorded in ppm on δ scale.

Microanalyses for C, H, and N were carried out on Perkin-Elmer 2400 analyzer (Perkin-Elmer, Norwalk, CT, USA) at the Microanalytical unit of Al Azhar University, Egypt and all compounds were within ± 0.4% of the theoretical values.

*p*-Methylthioacetophenone (**2**) and *p*-methylsulfonyl acetophenone (**3**) and 5-Un/substituted-2-(4-(methylsulfonyl) phenyl)-1*H*-indole (**5a-c**) were prepared according to a previous procedure [13]. The compounds were confirmed by matching their physical properties with the reported ones.

#### General procedure for synthesis of 5-substituted-2-(4-(methylsulfonyl)phenyl)-1H-indole-3-carbaldehyde 6a-c

A mixture of phosphorous oxychloride POCl_3_ (1.53 g, 10 mmol) and DMF (0.73 g, 10 mmol) was stirred for 30 min at room temperature, the solution of respective indole (1 mmol) in DMF (5 mL) was added slowly to the mixture which allowed to stir overnight. The reaction mixture was poured into ice-cold water and neutralized with 40% NaOH. The separated solid was filtered, dried and recrystallized from ethyl alcohol (yield: 70–80%).

##### 2-(4-(Methylsulfonyl)phenyl)-1H-indole-3-carbaldehyde (6a)

Yellow solid; Yield 70%; mp 232–235 ℃; IR (KBr, cm^−1^) 3205 (NH), 3065–3042 (CH aromatic), 2929–2871 (CH aliphatic), 1657 (C=O), 1305, 1150 (SO_2_); ^1^H NMR (DMSO-*d*_6_) δ (ppm): 3.21 (s, 3H, SO_2_CH_3_), 7.27–7.36 (m, 2H, indole H-5, H-6), 7.57 (d, 1H, *J* = 8 Hz, indole H-7), 8.08 (d, 2H, *J* = 8.4 Hz, phenyl H-2, H-6), 8.15 (d, 2H, *J* = 8.4 Hz, phenyl H-3, H-5), 8.26 (d, 1H, *J* = 7.6 Hz, indole H-4), 10.04 (s, 1H, aldehydic H), 12.64 (s, 1H, indole NH, D_2_O exchangeable). Anal. Calced for C_16_H_13_NO_3_S: C, 64.20; H, 4.38; N, 4.68. Found: C, 64.48; H, 4.40; N, 4.84.

##### 5-Methyl-2-(4-(methylsulfonyl)phenyl)-1H-indole-3-carbaldehyde (6b)

Brown solid; Yield 80%; mp 244–246 ℃; IR (KBr, cm^−1^) 3279 (NH), 3059–3029 (CH aromatic), 2927–2856 (CH aliphatic), 1670 (C=O), 1301, 1148 (SO_2_); ^1^H NMR (DMSO-*d*_6_) δ (ppm): 2.45 (s, 3H, CH_3_), 3.17 (s, 3H, SO_2_CH_3_), 7.17 (d, 1H, *J* = 8 Hz, indole H-6), 7.46 (d, 1H, *J* = 8 Hz, indole H-7), 8.06–8.14 (m, 5H, indole H-4, phenyl H-2, H-3, H-5, H-6), 10.00 (s, 1H, aldehydic H), 12.62 (s, 1H, indole NH, D_2_O exchangeable). Anal. Calced for C_17_H_15_NO_3_S: C, 65.16; H, 4.82; N, 4.47. Found: C, 65.27; H, 4.68; N, 4.52.

##### 5-Fluoro-2-(4-(methylsulfonyl)phenyl)-1H-indole-3-carbaldehyde (6c)

Yellow solid; Yield 72%; mp 195–197 ℃; IR (KBr, cm^−1^) 3320 (NH), 3064–3027 (CH aromatic), 2928–2853 (CH aliphatic), 1661 (C=O), 1302, 1146 (SO_2_); ^1^H NMR (DMSO-*d*_6_) δ (ppm): 3.18 (s, 3H, SO_2_CH_3_), 7.2 (d, 1H, *J* = 8 Hz, indole H-6), 7.58 (s, 1H, indole H-4), 7.91 (d, 1H, *J* = 9.6 Hz, indole H-7), 8.09 (d, 2H, *J* = 8.4 Hz, phenyl H-2, H-6), 8.14 (d, 2H, *J* = 8.4 Hz, phenyl H-3, H-5), 10.00 (s, 1H, aldehydic H), 12.92 (s, 1H, indole NH, D_2_O exchangeable). Anal. Calced for C_16_H_12_FNO_3_S: C, 60.56; H, 3.81; N, 4.41. Found: C, 60.73; H, 3.72; N, 4.62.

##### General procedure for synthesis of 5-substituted-3-((2-(4-substituted- phenyl)hydrazono) methyl)-2-(4-(methylsulfonyl)phenyl)-1H-indole 7a-k

A mixture of an ethanolic solution of respective indole-3-carbaldehyde derivative (**6a–c**) (1 mmol) and 4-substituted phenylhydrazine HCl (1 mmol) was heated under reflux for 4–6 h in the presence of a few drops of glacial acetic acid. After cooling, the reaction mixture was poured into ice-cold water and the separated solid was filtered, dried and recrystallized from methanol (yield: 73–92%).

##### 3-((2-(4-Fluorophenyl)hydrazono)methyl)-2-(4-(methylsulfonyl)phenyl)-1H-indole (7a)

Brown solid; Yield 73%; mp 204–206 ℃; IR (KBr, cm^−1^) 3282–3317 (indole NH, hydrazone NH), 3063 (CH aromatic), 2927–2843 (CH aliphatic), 1597 (C=N), 1302, 1148 (SO_2_); ^1^H NMR (DMSO-*d*_6_) δ (ppm): 3.26 (s, 3H, SO_2_CH_3_), 7.04–7.18 (m, 4H, phenyl hydrazone H-3, H-5, indole H-5, H-6), 7.44 (d, 1H, *J* = 8 Hz, indole H-4), 7.59 (d, 2H, *J* = 8.4 Hz, phenyl hydrazone H-2, H-6), 7.99 (d, 2H, *J* = 8.4 Hz, phenyl H-2, H-6), 8.12 (d, 2H, *J* = 8.4 Hz, phenyl H-3, H-5), 8.27 (s, 1H, CH), 8.4 (d, 1H, *J* = 8 Hz, indole H-7), 10.01 (s, 1H, hydrazone NH, D_2_O exchangeable), 11.79 (s, 1H, indole NH, D_2_O exchangeable); ^13^C NMR (DMSO-*d*_6_) δ (ppm): 43.0 (SO_2_CH_3_), 110.4, 111.9, 112.7, 115.2, 120.3, 125.6, 126.2, 128.0, 129.7, 132.1, 135.7, 136.4, 137.3, 140.2, 143.6 (CH=N), 154.7, 157.1. Anal. Calced for C_22_H_18_FN_3_O_2_S: C, 64.85; H, 4.45; N, 10.31. Found: C, 65.08; H, 4.33; N, 9.95.

##### 2-(4-(Methylsulfonyl)phenyl)-3-((2-(4-(methylsulfonyl)phenyl)hydrazono)methyl)-1H-indole (7b)

Yellow solid; Yield 85%; mp 228–230 ℃; IR (KBr, cm^−1^) 3262–3309 (indole NH, hydrazone NH), 3017 (CH aromatic), 2934–2863 (CH aliphatic), 1593 (C=N), 1299, 1150 (SO_2_); ^1^H NMR (DMSO-*d*_6_) δ (ppm): 3.11 (s, 3H, SO_2_CH_3_), 3.33 (s, 3H, SO_2_CH_3_), 7.17 (d, 2H, *J* = 8 Hz, phenyl hydrazone H-3, H-5), 7.24–7.33 (m, 2H, indole H-5, H-6), 7.51 (d, 1H, *J* = 8 Hz, indole H-4), 7.75 (d, 2H, *J* = 8 Hz, phenyl hydrazone H-2, H-6), 7.95 (d, 2H, *J* = 8 Hz, phenyl H-2, H-6), 8.13 (d, 2H, *J* = 8 Hz, phenyl H-3, H-5), 8.3 (s, 1H, CH), 8.4 (d, 1H, *J* = 8 Hz, indole H-7), 10.72 (s, 1H, hydrazone NH, D_2_O exchangeable), 11.98 (s, 1H, indole NH, D_2_O exchangeable); ^13^C NMR (DMSO-*d*_6_) δ (ppm): 43.9 (SO_2_CH_3_), 44.8 (SO_2_CH_3_), 110.5, 111.2, 112.2, 121.5, 122.7, 124.1, 125.7, 127.9, 128.7, 129.5, 130.2, 136.8, 137.3, 137.6, 137.8, 140.6, 149.8 (CH = N). Anal. Calced for C_23_H_21_N_3_O_4_S_2_: C, 59.08; H, 4.53; N, 8.99. Found: C, 59.27; H, 4.68; N, 9.12.

##### 4-(2-((2-(4-(Methylsulfonyl)phenyl)-1H-indol-3-yl)methylene)hydrazinyl)benzene sulfonamide (7c)

Yellow solid; Yield 83%; mp 203–204 ℃; IR (KBr, cm^−1^) 3298–3325 (NH_2_, indole NH, hydrazone NH), 3014 (CH aromatic), 2924–2853 (CH aliphatic), 1593 (C=N), 1276, 1089 (SO_2_); ^1^H NMR (DMSO-*d*_6_) δ (ppm): 3.11 (s, 3H, SO_2_CH_3_), 7.17 (d, 2H, *J* = 8 Hz, phenyl hydrazone H-3, H-5), 7.24–7.33 (m, 2H, indole H-5, H-6), 7.5 (d, 1H, *J* = 8 Hz, indole H-4), 7.75 (d, 2H, *J* = 8 Hz, phenyl hydrazone H-2, H-6), 7.95 (d, 2H, *J* = 8 Hz, phenyl H-2, H-6), 8.13 (d, 2H, *J* = 8 Hz, phenyl H-3, H-5), 8.36 (s, 1H, CH), 8.39 (d, 1H, *J* = 8 Hz, indole H-7), 10.71 (s, 1H, hydrazone NH, D_2_O exchangeable), 11.97 (s, 1H, indole NH, D_2_O exchangeable), NH_2_ not distinguished; ^13^C NMR (DMSO-*d*_6_) δ (ppm): 43.9 (SO_2_CH_3_), 110.5, 111.2, 112.2, 121.5, 122.7, 124.1, 125.7, 127.9, 128.7, 129.5, 130.2, 136.8, 137.3, 137.6, 137.8, 140.6, 149.8 (CH=N). Anal. Calced for C_22_H_20_N_4_O_4_S_2_: C, 56.39; H, 4.30; N, 11.96. Found: C, 56.45; H, 4.17; N, 12.28.

##### 5-Methyl-2-(4-(methylsulfonyl)phenyl)-3-((2-(4-(methylsulfonyl)phenyl) hydraz-ono) methyl)-1H-indole (7d)

Brown solid; Yield 85%; mp 262–264 ℃; IR (KBr, cm^−1^) 3319–3340 (indole NH, hydrazone NH), 3023 (CH aromatic), 2932–2856 (CH aliphatic), 1595 (C=N), 1300, 1140 (SO_2_); ^1^H NMR (DMSO-*d*_6_) δ (ppm): 2.55 (s, 3H, CH_3_), 3.11 (s, 3H, SO_2_CH_3_), 3.31 (s, 3H, SO_2_CH_3_), 7.12–7.18 (m, 3H, indole H-6, phenyl hydrazone H-3, H-5), 7.4 (d, 1H, *J* = 8.4 Hz, indole H-7), 7.76 (d, 2H, *J* = 8.4 Hz, phenyl hydrazone H-2, H-6), 7.92 (d, 2H, *J* = 8 Hz, phenyl H-2, H-6), 8.11 (d, 2H, *J* = 8 Hz, phenyl H-3, H-5), 8.35 (s, 1H, CH), 8.18 (s, 1H, indole H-4), 10.71 (s, 1H, hydrazone NH, D_2_O exchangeable), 11.88 (s, 1H, indole NH, D_2_O exchangeable); ^13^C NMR (DMSO-*d*_6_) δ (ppm): 22.0 (CH_3_), 44.0 (SO_2_CH_3_), 44.8 (SO_2_CH_3_), 110.1, 111.2, 111.9, 122.2, 125.6, 126.0, 126.8, 127.8, 128.7, 129.5, 129.8, 130.1, 135.7, 136.9, 137.8, 140.6, 149.8 (CH=N). Anal. Calced for C_24_H_23_N_3_O_4_S_2_: C, 59.86; H, 4.81; N, 8.73. Found: C, 59.67; H, 4.82; N, 8.97.

##### 4-(2-((5-Methyl-2-(4-(methylsulfonyl)phenyl)-1H-indol-3-yl)methylene) hydrazine-yl) benzenesulfonamide (7e)

Yellow solid; Yield 87%; mp 186–188 ℃; IR (KBr, cm^−1^) 3300–3341 (NH_2_, indole NH, hydrazone NH), 3023 (CH aromatic), 2927–2854 (CH aliphatic), 1595 (C=N), 1300, 1130 (SO_2_); ^1^H NMR (DMSO-*d*_6_) δ (ppm): 3.1 (s, 3H, CH_3_), 3.33 (s, 3H, SO_2_CH_3_), 7.12–7.18 (m, 4H, phenyl hydrazone H-3, H-5, indole H-4, H-6), 7.39 (d, 1H, *J* = 8 Hz, indole H-7), 7.76 (d, 2H, *J* = 8 Hz, phenyl hydrazone H-2, H-6), 7.93 (d, 2H, *J* = 8 Hz, phenyl H-2, H-6), 8.12 (d, 2H, *J* = 8 Hz, phenyl H-3, H-5), 8.18 (s, 2H, NH_2_, D_2_O exchangeable), 8.35 (s, 1H, CH), 10.7 (s, 1H, hydrazone NH, D_2_O exchangeable), 11.88 (s, 1H, indole NH, D_2_O exchangeable); ^13^C NMR (DMSO-*d*_6_) δ (ppm): 22.0 (CH_3_), 43.9 (SO_2_CH_3_), 110.1, 111.2, 111.9, 122.3, 125.6, 125.9, 127.9, 128.6, 129.5, 129.8, 130.1, 135.7, 136.9, 137.8, 137.8, 140.5, 149.8 (CH=N). Anal. Calced for C_23_H_22_N_4_O_4_S_2_: C, 57.24; H, 4.60; N, 11.61. Found: C, 57.56; H, 4.53; N, 11.89.

##### 3-((2-(4-Fluorophenyl)hydrazono)methyl)-5-methyl-2-(4-(methylsulfonyl)phenyl)-1H-indole (7f)

Yellow solid; Yield 80%; mp 159–161 ℃; IR (KBr, cm^−1^) 3250–3307 (indole NH, hydrazone NH), 3065 (CH aromatic), 2928–2859 (CH aliphatic), 1597 (C=N), 1300, 1146 (SO_2_); ^1^H NMR (DMSO-*d*_6_) δ (ppm): 2.49 (s, 3H, CH_3_), 3.4 (s, 3H, SO_2_CH_3_), 7.02 (d, 2H, *J* = 8.4 Hz, phenyl hydrazone H-3, H-5), 7.04–7.1 (m, 3H, phenyl hydrazone H-2, H-6, indole H-7), 7.37 (d, 1H, *J* = 8 Hz, indole H-6), 7.92 (d, 2H, *J* = 8 Hz, phenyl H-2, H-6), 8.1 (d, 2H, *J* = 8 Hz, phenyl H-3, H-5), 8.18 (s, 1H, indole H-4),8.25 (s, 1H, CH), 10.03 (s, 1H, hydrazone NH, D_2_O exchangeable), 11.75 (s, 1H, indole NH, D_2_O exchangeable); ^13^C NMR (DMSO-*d*_6_) δ (ppm): 22.6 (CH_3_), 43.6 (SO_2_CH_3_), 110.4, 111.5, 112.1, 116.2, 122.3, 125.4, 126.1, 127.8, 129.8, 134.6, 135.7, 136.4, 137.2, 140.2, 143.1 (CH=N), 154.7, 157.0. Anal. Calced for C_23_H_20_FN_3_O_2_S: C, 65.54; H, 4.78; N, 9.97. Found: C, 65.6; H, 4.6; N, 9.94.

##### 5-Methyl-2-(4-(methylsulfonyl)phenyl)-3-((2-(p-tolyl)hydrazono)methyl)-1H-indole (7 g)

Brown solid; Yield 84%; mp 166-168 ℃; IR (KBr, cm^−1^) 3214–3306 (indole NH, hydrazone NH), 3023 (CH aromatic), 2926–28,658 (CH aliphatic), 1598 (C=N), 1302, 1149 (SO_2_); ^1^H NMR (DMSO-*d*_6_) δ (ppm): 2.32 (s, 3H, CH_3_), 2.48 (s, 3H, CH_3_), 3.25 (s, 3H, SO_2_CH_3_), 6.95–7.07 (m, 3H, indole H-6, phenyl hydrazone H-3, H-5), 7.39 (d, 1H, *J* = 8.4 Hz, indole H-7), 7.62 (s, 2H, NH_2_, D_2_O exchangeable), 7.80 (d, 2H, *J* = 8.4 Hz, phenyl hydrazone H-2, H-6), 7.92 (d, 2H, *J* = 8.4 Hz, phenyl H-2, H-6), 8.10 (d, 2H, *J* = 8.4 Hz, phenyl H-3, H-5), 8.2 (s, 1H, CH), 8.25 (s, 1H, indole H-4), 9.91 (s, 1H, hydrazone NH, D_2_O exchangeable), 11.71 (s, 1H, indole NH, D_2_O exchangeable); ^13^C NMR (DMSO-*d*_6_) δ (ppm): 20.5 (CH_3_), 22.1 (CH_3_), 44.0 (SO_2_CH_3_), 111.0, 111.9, 120.5, 122.5, 125.6, 126.1, 126.8, 127.2, 127.8, 128.1, 129.6, 130.0, 135.2, 136.1, 137.7, 140.7, 144.1 (CH=N). Anal. Calced for C_24_H_23_N_3_O_2_S: C, 69.04; H, 5.55; N, 10.06. Found: C, 68.82; H, 5.68; N, 10.32.

##### 5-Fluoro-2-(4-(methylsulfonyl)phenyl)-3-((2-(4-(methylsulfonyl)phenyl) hydraz-ono)methyl)-1H-indole (7h)

Bale yellow solid; Yield 92%; mp 187–188 ℃; IR (KBr, cm^−1^) 3265–3337 (indole NH, hydrazone NH),3025 (CH aromatic), 2925–2854 (CH aliphatic), 1593 (C=N), 1321, 1140 (SO_2_); ^1^H NMR (DMSO-*d*_6_) δ (ppm): 3.12 (s, 3H, SO_2_CH_3_), 3.34 (s, 3H, SO_2_CH_3_), 7.15–7.20 (m, 3H, phenyl hydrazone H-3, H-5, indole H-6), 7.51 (s, 1H, indole H-4), 7.77 (d, 2H, *J* = 8 Hz, phenyl hydrazone H-2, H-6), 7.95 (d, 2H, *J* = 8 Hz, phenyl H-2, H-6), 8.04 (d, 1H, *J* = 8 Hz, indole H-7), 8.14 (d, 2H, *J* = 8 Hz, phenyl H-3, H-5), 8.34 (s, 1H, CH), 10.73 (s, 1H, hydrazone NH, D_2_O exchangeable), 12.11 (s, 1H, indole NH, D_2_O exchangeable); ^13^C NMR (DMSO-*d*_6_) δ (ppm): 44.0 (SO_2_CH_3_), 44.8 (SO_2_CH_3_), 107.0, 111.3, 112.1, 113.4, 125.9, 127.9, 129.0, 129.5, 130.2, 134.0, 136.5, 137.4, 139.4, 140.9, 149.7 (CH=N), 157.2, 159.5. Anal. Calced for C_23_H_20_FN_3_O_4_S_2_: C, 56.89; H, 4.15; N, 8.65. Found: C, 57.17; H, 4.23; N, 8.58.

##### 4-(2-((5-Fluoro-2-(4-(methylsulfonyl)phenyl)-1H-indol-3yl)methylene)hydrazinyl) benzene sulfonamide (7i)

Yellow solid; Yield 82%; mp 212–214 ℃; IR (KBr, cm^−1^) 3260–3315 (NH_2_, indole NH, hydrazone NH), 3026 (CH aromatic), 2927 (CH aliphatic), 1594 (C=N), 1295, 1140 (SO_2_); ^1^H NMR (DMSO-*d*_6_) δ (ppm): 3.24 (s, 3H, SO_2_CH_3_), 7.14 (d, 2H, *J* = 8 Hz, phenyl hydrazone H-3, H-5), 7.51 (s, 1H, indole H-4), 7.67 (d, 1H, *J* = 8 Hz, indole H-6), 7.76 (d, 2H, *J* = 8 Hz, phenyl hydrazone H-2, H-6), 7.91 (s, 2H, NH_2_, D_2_O exchangeable), 7.95 (d, 2H, *J* = 8 Hz, phenyl H-2, H-6), 8.03 (d, 1H, *J* = 8 Hz, indole H-7), 8.13 (d, 2H, *J* = 8 Hz, phenyl H-3, H-5), 8.34 (s, 1H, CH), 10.62 (s, 1H, hydrazone NH, D_2_O exchangeable), 11.99 (s, 1H, indole NH, D_2_O exchangeable); ^13^C NMR (DMSO-*d*_6_) δ (ppm): 44.0 (SO_2_CH_3_), 107.0, 110.6, 112.4, 113.5, 125.9, 127.5, 129.0, 129.2, 130.2, 134.0, 135.5, 136.4, 139.4, 140.8, 141.0, 149.7 (CH=N), 157.2. Anal. Calced for C_22_H_19_FN_4_O_4_S_2_: C, 54.31; H, 3.94; N, 11.52. Found: C, 54.67; H, 3.82; N, 11.73.

##### 5-Fluoro-3-((2-(4-fluorophenyl)hydrazono)methyl)-2-(4-(methylsulfonyl)phenyl)-1H-indole (7j)

Yellow solid; Yield 82%; mp 200–202 ℃; IR (KBr, cm^−1^) 3217–3250 (indole NH, hydrazone NH), 3065 (CH aromatic), 2928–2863 (CH aliphatic), 1597 (C=N), 1302, 1145 (SO_2_); ^1^H NMR (DMSO-*d*_6_) δ (ppm): 3.33 (s, 3H, SO_2_CH_3_), 7.01 (d, 2H, *J* = 8 Hz, phenyl hydrazone H-3, H-5), 7.09–7.17 (m, 3H, phenylhydrazone H-2, H-6, indole H-6), 7.5 (s, 1H, indole H-4), 7.94 (d, 2H, *J* = 8 Hz, phenyl H-2, H-6), 8.03 (d, 1H, *J* = 8 Hz, indole H-7), 8.12 (d, 2H, *J* = 8 Hz, phenyl H-3, H-5), 8.25 (s, 1H, CH), 10.09 (s, 1H, hydrazone NH, D_2_O exchangeable), 11.99 (s, 1H, indole NH, D_2_O exchangeable); ^13^C NMR (DMSO-*d*_6_) δ (ppm): 44.0 (SO_2_CH_3_), 107.3, 111.1, 112.0, 112.7, 113.0, 116.3, 125.9, 127.9, 130.1, 134.0, 136.7, 137.9, 140.6, 142.9 (CH=N), 154.7, 157.0, 159.3. Anal. Calced for C_22_H_17_F_2_N_3_O_2_S: C, 62.11; H, 4.03; N, 9.88. Found: C, 62.32; H, 4.11; N, 10.16.

##### 5-Fluoro-2-(4-(methylsulfonyl)phenyl)-3-((2-(p-tolyl)hydrazono)methyl)-1H-indole (7k)

Brown solid; Yield 75%; mp 151–153 ℃; IR (KBr, cm^−1^) 3220–3270 (indole NH, hydrazone NH),3034 (CH aromatic), 2927, 2860 (CH aliphatic), 1597 (C=N), 1303, 1146 (SO_2_); ^1^H NMR (DMSO-*d*_6_) δ (ppm): 2.23 (s, 3H, CH_3_), 3.33 (s, 3H, SO_2_CH_3_), 6.94 (d, 2H, *J* = 12 Hz, phenyl hydrazone H-3, H-5), 7.07 (d, 2H, *J* = 12 Hz, phenyl hydrazone H-2, H-6),7.15 (d,1H, *J* = 8 Hz, indole H-6), 7.48 (s, 1H, indole H-4), 7.94 (d, 2H, *J* = 8 Hz, phenyl H-2, H-6), 8.05 (d, 1H, *J* = 12 Hz, indole H-7), 8.12 (d, 2H, *J* = 8 Hz, phenyl H-3, H-5), 8.24 (s, 1H, CH), 10.01 (s, 1H, hydrazone NH, D_2_O exchangable), 11.96 (s, 1H, indole NH, D_2_O exchangable; ^13^C NMR (DMSO-*d*_6_) δ (ppm): 20.7 (CH_3_), 43.9 (SO_2_CH_3_), 105.3, 111.4, 112.2, 113.3, 125.9, 126.9, 127.3, 128.2, 129.9, 134.0, 134.8, 137.7, 137.9, 140.5, 144.0 (CH = N), 157.0, 159.3. Anal. Calced for C_23_H_20_FN_3_O_2_S: C, 65.54; H, 4.78; N, 9.97 Found: C, 65.70; H, 5.03; N, 10.14.

##### General procedure for synthesis of 2-(5-substituted-2-(4-(methylsulfonyl)phenyl)-1H-indol-3-yl)-6-chloro-1H-benzo[d]imidazole 8a-c

A mixture of 4-chloro phenylene diamine (0.142 g, 1 mmol), sodium metabisulfite (0.19 g, 1 mmol) and respective indole-3-carbaldehyde derivative (**6a–c**) (1 mmol) in DMF was heated under reflux for 6 h. After cooling, the reaction mixture was poured into ice cold water and the separated solid was filtered, dried and recrystallized from ethanol (yield: 60–70%).

##### 5-Chloro-2-(2-(4-(methylsulfonyl)phenyl)-1H-indol-3-yl)-1H-benzo[d]imidazole (8a)

Yellow solid; Yield 60%; mp 210–212 ℃; IR (KBr, cm^−1^) 3285–3382 (indole NH, benzimidazole NH), 3065–3021 (CH aromatic), 2926–2853 (CH aliphatic), 1660 (benzimidazole C=N), 1301, 1149 (SO_2_); ^1^H NMR (DMSO-*d*_6_) δ (ppm): 3.27 (s, 3H, SO_2_CH_3_), 7.19–7.22 (m, 2H, indole H-5, benzimidazole H-6), 7.3 (t, 1H, *J* = 7.4 Hz, indole H-6), 7.47 (s, 1H, benzimidazole H-4), 7.55 (d, 1H, *J* = 8 Hz, benzimidazole H-7), 7.69 (s, 1H, indole H-7), 7.89–7.92 (m, 3H, phenyl H-2, H-6, indole H-4), 7.99 (d, 2H, *J* = 8.4 Hz, phenyl H-3, H-5), 12.18 (s, 1H, indole NH, D_2_O exchangeable), 12.45 (s, 1H, benzimidazole NH, D_2_O exchangeable); ^13^C NMR (DMSO-*d*_6_) δ (ppm): 43.8 (SO_2_CH_3_), 105.1, 112.4, 116.4, 116.8, 117.5, 120.7, 121.2, 122.5, 123.8, 127.7, 128.0, 129.5, 131.5, 135.3, 136.2, 136.8, 136.9, 140.5, 145.8. Anal. Calced for C_22_H_16_ClN_3_O_2_S: C, 62.63; H, 3.82; N, 9.96. Found: C, 62.89; H, 3.68; N, 10.24.

##### 5-Chloro-2-(5-fluoro-2-(4-(methylsulfonyl)phenyl)-1H-indol-3-yl)-1H-benzo[d] imidazole (8b)

Pale yellow; Yield 67%; mp 202–204 ℃; IR (KBr, cm^−1^) 3348–3360 (indole NH, benzimidazole NH), 3008–3063 (CH aromatic), 2854–2928 (CH aliphatic), 1659 (benzimidazole C=N), 1300, 1148 (SO_2_); ^1^H NMR (DMSO-*d*_6_) δ (ppm): 3.29 (s, 3H, SO_2_CH_3_), 7.13–7.22 (m, 2H, indole H-6, benzimidazole H-6), 7.46 (d, 1H, *J* = 8 Hz, benzimidazole H-7), 7.55 (s, 1H, indole H-4), 7.66–7.72 (m, 2H, benzimidazole H-4, indole H-7), 7.91 (d, 2H, *J* = 8 Hz, phenyl H-2, H-6), 8.02 (d, 2H, *J* = 8 Hz, phenyl H-3, H-5), 12.25 (s, 1H, indole NH, D_2_O exchangeable), 12.37 (s, 1H, benzimidazole NH, D_2_O exchangeable); ^13^C NMR (DMSO-*d*_6_) δ (ppm): 43.9 (SO_2_CH_3_), 105.4, 106.3, 112.1, 113.6, 117.3, 118.3, 122.0, 123.8, 125.2, 127.7, 129.7, 133.5, 136.6, 138.1, 140.4, 140.9, 141.9, 146.3, 149.0. Anal. Calced for C_22_H_15_ClFN_3_O_2_S: C, 60.07; H, 3.44; N, 9.55. Found: C, 60.31; H, 3.20; N, 9.79.

##### 5-Chloro-2-(5-methyl-2-(4-(methylsulfonyl)phenyl)-1H-indol-3-yl)-1H-benzo[d]imidazole (8c)

Yellow solid; Yield 70%; mp 217–219 ℃; IR (KBr, cm^−1^) 3272–3322 (indole NH, benzimidazole NH), 3192, 3072 (CH aromatic), 2927, 2857 (CH aliphatic), 1620 (benzimidazole C=N), 1301, 1149 (SO_2_); ^1^H NMR (DMSO-*d*_6_) δ (ppm): 2.43 (s, 3H, CH_3_),3.27 (s, 3H, SO_2_CH_3_), 7.12 (d, 1H, *J* = 8.4 Hz, indole H-6), 7.2 (d, 1H, *J* = 8.4 Hz, benzimidazole H-6), 7.43–7.47 (m, 2H, indole H-7, benzimidazole H-7), 7.68–7.71 (m, 2H, indole H-4, benzimidazole H-4), 7.88 (d, 2H, *J* = 8.4 Hz, phenyl H-2, H-6), 7.98 (d, 2H, *J* = 8.4 Hz, phenyl H-3, H-5), 12.04 (s, 1H, indole NH, D_2_O exchangeable), 12.45 (s, 1H, benzimidazole NH, D_2_O exchangeable); ^13^C NMR (DMSO-*d*_6_) δ (ppm): 21.7 (CH_3_), 43.8 (SO_2_CH_3_), 104.7, 112.1, 113.2, 118.4, 118.6, 120.1, 122.7, 125.5, 126.2, 127.6, 128.3, 129.4, 130.0, 134.3, 135.2, 136.1, 137.0, 140.4, 145.7. Anal. Calced for C_23_H_18_ClN_3_O_2_S: C, 63.37; H, 4.16; N, 9.64. Found: C, 63.24; H, 4.25; N, 9.88.

##### General procedure for synthesis of 5-un/substituted-2-(4-(methylsulfonyl)phenyl)-1H-indole-3-carbaldehyde oxime 9a–c

A mixture of an ethanolic solution of respective indole-3-carbaldehyde derivative (**6a–c**) (1 mmol) and hydroxylamine HCl (0.08 g, 1 mmol) was heated under reflux for 4–6 h in the presence of a few drops of pyridine. After cooling, the reaction mixture was poured into ice-cold water and the separated solid was filtered, dried and recrystallized from ethanol (yield: 55–70%).

##### 2-(4-(Methylsulfonyl)phenyl)-1H-indole-3-carbaldehyde oxime (9a)

Yellow solid; Yield 62%; mp 199–201 ℃; IR (KBr, cm^−1^) 3282–3385 (indole NH, OH), 3010–3028 (CH aromatic), 2928–2951 (CH aliphatic), 1596 (C=N), 1302, 1146 (SO_2_); ^1^H NMR (DMSO-*d*_6_) δ (ppm): 3.31 (s, 3H, SO_2_CH_3_), 7.16–7.26 (m, 2H, indole H-5, H-6), 7.48 (d, 1H, *J* = 8 Hz, indole H-7), 7.89 (d, 2H, *J* = 8 Hz, phenyl H-2, H-6), 8.10–8.12 (m, 3H, phenyl H-3, H-5, indole H-4), 8.32 (s, 1H, CH), 10.89 (s, 1H, OH, D_2_O exchangeable), 11.96 (s, 1H, indole NH, D_2_O exchangeable); ^13^C NMR (DMSO-*d*_6_) δ (ppm): 44.2 (SO_2_CH_3_), 106.4, 112.7, 122.4, 125.7, 126.2, 127.9, 129.0, 129.9, 135.6, 136.7, 137.7, 140.7, 143.3 (CH=N). Anal. Calced for C_16_H_14_N_2_O_3_S: C, 61.13; H, 4.49; N, 8.91. Found: C, 61.48; H, 4.61; N, 8.62.

##### 5-Fluoro-2-(4-(methylsulfonyl)phenyl)-1H-indole-3-carbaldehyde oxime (9b)

Yellow solid; Yield 55%; mp 226–228 ℃; IR (KBr, cm^−1^) 3366–3463 (indole NH, OH), 3013–3029 (CH aromatic), 2918–2997 (CH aliphatic), 1598 (C=N), 1298, 1143 (SO_2_); ^1^H NMR (DMSO-*d*_6_) δ (ppm): 3.3 (s, 3H, SO_2_CH_3_), 7.12 (d, 1H, *J* = 8 Hz, indole H-6), 7.48 (s, 1H, indole H-4), 7.8 (d, 1H, *J* = 8 Hz, indole H-7), 7.89 (d, 2H, *J* = 8 Hz, phenyl H-2, H-6), 8.11 (d, 2H, *J* = 8 Hz, phenyl H-3, H-5), 8.31 (s, 1H, CH), 10.89 (s, 1H, OH, D_2_O exchangeable), 12.04 (s, 1H, indole NH, D_2_O exchangeable); ^13^C NMR (DMSO-*d*_6_) δ (ppm): 43.9 (SO_2_CH_3_), 107.2, 112.2, 113.3, 126.2, 128.0, 129.5, 133.8, 136.3, 139.3, 140.5, 144.0 (CH=N), 157.1, 159.4. Anal. Calced for C_16_H_13_FN_2_O_3_S: C, 57.82; H, 3.94; N, 8.43. Found: C, 57.58; H, 4.06; N, 8.75.

##### 5-Methyl-2-(4-(methylsulfonyl)phenyl)-1H-indole-3-carbaldehyde oxime (9c)

Yellow solid; Yield 70%; mp 212–214 ℃  ℃; IR (KBr, cm^−1^) 3362 (indole NH, OH), 3025–3060 (CH aromatic), 2857–2928 (CH aliphatic), 1597 (C=N), 1300, 1145 (SO_2_); ^1^H NMR (DMSO-*d*_6_) δ (ppm): 2.42 (s, 3H, CH_3_), 3.29 (s, 3H, SO_2_CH_3_), 7.09 (d, 1H, *J* = 8 Hz, indole H-7), 7.36 (d, 1H, *J* = 8 Hz, indole H-6), 7.86 (d, 2H, *J* = 8 Hz, phenyl H-2, H-6),7.94 (s, 1H, indole H-4), 8.09 (d, 2H, *J* = 8 Hz, phenyl H-3, H-5), 8.31 (s, 1H, CH), 10.8 (s, 1H, OH, D_2_O exchangeable), 11.79 (s, 1H, indole NH, D_2_O exchangeable); ^13^C NMR (DMSO-*d*_6_) δ (ppm): 21.7 (CH_3_), 44.0 (SO_2_CH_3_), 107.4, 111.8, 122.2, 125.4, 126.2, 127.7, 129.0, 129.9, 135.5, 136.7, 137.7, 140.5, 144.3 (CH=N). Anal. Calced for C_17_H_16_N_2_O_3_S: C, 62.18; H, 4.91; N, 8.53. Found: C, 62.42; H, 4.83; N, 8.79.

### Biological evaluation

#### Antimicrobial and antifungal activities

The antimicrobial and antifungal screening was performed according to CO-ADD (The Community for Antimicrobial Drug Discovery) procedures [27].

#### COX-1/COX-2 inhibition colorimetric assay

Measurement of the ability of the synthesized compounds to inhibit COX isozymes by using colorimetric COX (ovine) inhibitor screening assay kit (Kit catalog number 760111, Cayman Chemical, Ann Arbor, MI, USA) following the manufacturer’s instructions and as mentioned before [28].

#### Carrageenan-induced rat edema assay

Pretreatment of rats with compounds **7a–k, 8a–c,** and **9a–c** before injection with carrageenan in rat paw which induces inflammation and then the percentage of paw edema reduction was measured after certain hours according to previously reported procedures [29].

#### In vitro nitric oxide release assay

Different solutions of the tested compounds **9a–c** in DMF were diluted using phosphate buffer (pH 7.4) till a final concentration of 100 µM (test solutions). To 100 µl of different test solutions, 100 µl of N-acetyl cysteine solution was added and the obtained solution was kept in an incubator at 37 °C (treated solutions). The solutions were treated similarly as for a nitrite standard solution with Griess reagent components, 100 µl of sulphanilamide solution was added to each tube of the treated solution, the mixture was left at 25 °C for 5–10 min, protected from light. To this mixture 100 µl of the NED solution was added, the mixture was again left for 5–10 min at 25 °C, protected from light.

The absorbance of the formed purple color, if any, was measured within 30 min at λ 546 nm, a blank experiment was performed under the same conditions, the procedure was repeated three times for each tested compound and the average absorbance values were calculated. The corresponding concentration of nitrite was determined by comparison to the nitrite standard calibration curve and the amount of NO released (revealed by the corresponding nitrite concentration) was calculated as a percentage of moles of NO released from 1 mol of the tested compounds.

#### Molecular modeling and docking

Molecular modeling studies were performed by using Molecular Operating Environment MOE version 2018.0101. Structures of **7b, 7h,** and **7i** were built in MOE. The X-ray crystal structure of celecoxib bound to the COX-2 (PDB: ID 3LN1) active site was obtained from the protein data bank at research collaboration for Structural Bioinformatics (RSCB) protein database [PDB].

Preparation of the enzyme for docking by removing the Co-crystallized ligand and water molecules then the enzyme was 3D protonated, in which hydrogen atoms were added to their standard geometry. The conformers generated were docked into the COX-2 receptor with MOE-dock using the triangle matcher placement method and the GBVI/WSA dG scoring function.

A molecular mechanics force field refinement was carried out on the top 30 poses generated. Celecoxib was redocked into the active site of 3LN1 to validate the docking protocol. Amino acid interactions and the hydrogen bond lengths were summarized in (Table 6).

## Data Availability

The data sets and samples of the compounds used during the current study are available from the corresponding author on reasonable request.

## Abbreviations

*A. baumannii*: *Acinetobacter baumannii*
*C. albicans*: *Candida albicans*
*C. neoformans*: *Cryptococcus neoformans var. grubii*
*E. coli*: *Escherichia coli*
GI_50_: Growth Inhibition of 50%
*K. pneumonia*: *Klebsilla pneumonia*
MIC: Minimum Inhibitory Concentration
MOE: Molecular Operating Environment software
*MRSA*: *Methicillin*-*resistant Staphylococcus aureus*
*P. aeruginosa*: *Pseudomonas aeruginosa*
SI: Selectivity index

## References

[1] Akbas E, Berber I (2005). Antibacterial and antifungal activities of new pyrazolo [3, 4-*d*] pyridazin derivatives. Eur J Med Chem.

[2] Alagarsamy V, Meena S, Ramseshu K, Solomon V, Thirumurugan K, Dhanabal K, Murugan M (2006). Synthesis, analgesic, anti-inflammatory, ulcerogenic index and antibacterial activities of novel 2-methylthio-3-substituted-5, 6, 7, 8-tetrahydrobenzo (*b*) thieno [2, 3-*d*] pyrimidin-4 (3*H*)-ones. Eur J Med Chem.

[3] Bekhit AA, Farghaly AM, Shafik RM, Elsemary MM, Bekhit AE, Guemei AA, El-Shoukrofy MS, Ibrahim TM (2018). Synthesis, biological evaluation and molecular modeling of novel thienopyrimidinone and triazolothienopyrimidinone derivatives as dual anti-inflammatory antimicrobial agents. Bioorg Chem.

[4] Abdelall EK, Lamie PF, Ali WA (2016). Cyclooxygenase-2 and 15-lipoxygenase inhibition, synthesis, anti-inflammatory activity and ulcer liability of new celecoxib analogues: determination of region-specific pyrazole ring formation by NOESY. Bioorg Med Chem Lett.

[5] Zarghi A, Najafnia L, Daraee B, Dadrass OG, Hedayati M (2007). Synthesis of 2, 3-diaryl-1, 3-thiazolidine-4-one derivatives as selective cyclooxygenase (COX-2) inhibitors. Bioorg Med Chem Lett.

[6] Zebardast T, Zarghi A, Daraie B, Hedayati M, Dadrass OG (2009). Design and synthesis of 3-alkyl-2-aryl-1, 3-thiazinan-4-one derivatives as selective cyclooxygenase (COX-2) inhibitors. Bioorg Med Chem Lett.

[7] Abdellatif KR, Abdelall EK, Fadaly WA, Kamel GM (2016). Synthesis, cyclooxygenase inhibition, anti-inflammatory evaluation and ulcerogenic liability of new 1, 3, 5-triarylpyrazoline and 1, 5-diarylpyrazole derivatives as selective COX-2 inhibitors. Bioorg Med Chem Lett.

[8] Grosser T, Ricciotti E, FitzGerald GA (2017). The cardiovascular pharmacology of nonsteroidal anti-inflammatory drugs. Trends Pharmacol Sci.

[9] Elshemy HA, Abdelall EK, Azouz AA, Moawad A, Ali WA, Safwat NM (2017). Synthesis, anti-inflammatory, cyclooxygenases inhibitions assays and histopathological study of poly-substituted 1, 3, 5-triazines: confirmation of regiospecific pyrazole cyclization by HMBC. Eur J Med Chem.

[10] Patrono C, Baigent C (2017). Coxibs, traditional NSAIDs, and cardiovascular safety post-precision: what we thought we knew then and what we think we know now. Clin Pharmacol Ther.

[11] Smyth EM (2010). Thromboxane and the thromboxane receptor in cardiovascular disease. Clinical Lipidology.

[12] Xu S, Wang G, Lin Y, Zhang Y, Pei L, Yao H, Hu M, Qiu Y, Huang Z, Zhang Y (2016). Novel anticancer oridonin derivatives possessing a diazen-1-ium-1, 2-diolate nitric oxide donor moiety: design, synthesis, biological evaluation and nitric oxide release studies. Bioorg Med Chem Lett.

[13] Shaker AM, Abdelall EK, Abdellatif KR, Abdel-Rahman HM (2018). Design, synthesis, and biological evaluation of 2-(4-(methylsulfonyl) phenyl) indole derivatives with promising COX-2 inhibitory activity. J Appl Pharm Sci.

[14] Abdellatif KR, Abdelall EK, Bakr RB (2017). Nitric oxide-NASIDS donor prodrugs as hybrid safe anti-inflammatory agents. Curr Top Med Chem.

[15] Popiołek Ł (2017). Hydrazide–hydrazones as potential antimicrobial agents: overview of the literature since 2010. Med Chem Res.

[16] Özkay Y, Tunalı Y, Karaca H, Işıkdağ İ (2011). Antimicrobial activity of a new series of benzimidazole derivatives. Arch Pharm Res.

[17] Singh N, Pandurangan A, Rana K, Anand P, Ahamad A, Tiwari AK (2012). Benzimidazole: a short review of their antimicrobial activities. Int Curr Pharm J.

[18] Verma G, Marella A, Shaquiquzzaman M, Akhtar M, Ali MR, Alam MM (2014). A review exploring biological activities of hydrazones. J Pharm Bioallied Sci.

[19] Saini D, Gupta M (2018). Hydrazones as potential anticancer agents: an update. Asian J Pharm Pharmacol.

[20] Kaur J, Bhardwaj A, Huang Z, Knaus EE (2012). N-1 and C-3 substituted indole Schiff bases as selective COX-2 inhibitors: synthesis and biological evaluation. Bioorg Med Chem Lett.

[21] Bandgar BP, Sarangdhar RJ, Viswakarma S, Ahamed FA (2011). Synthesis and biological evaluation of orally active prodrugs of indomethacin. J Med Chem.

[22] Unsal-Tan O, Ozadali K, Piskin K, Balkan A (2012). Molecular modeling, synthesis and screening of some new 4-thiazolidinone derivatives with promising selective COX-2 inhibitory activity. Eur J Med Chem.

[23] El-Sherief HA, Abuo-Rahma GE-DA, Shoman ME, Beshr EA, Abdel-baky RM (2017). Design and synthesis of new coumarin–chalcone/NO hybrids of potential biological activity. Med Chem Res.

[24] Abuo-Rahma GE-DA, Abdel-Aziz M, Beshr EA, Ali TF (2014). 1, 2, 4-Triazole/oxime hybrids as new strategy for nitric oxide donors: synthesis, anti-inflammatory, ulceroginicity and antiproliferative activities. Eur J Med Chem.

[25] Habeeb AG, Praveen Rao P, Knaus EE (2001). Design and synthesis of celecoxib and rofecoxib analogues as selective cyclooxygenase-2 (COX-2) inhibitors: replacement of sulfonamide and methylsulfonyl pharmacophores by an azido bioisostere. J Med Chem.

[26] Wang JL, Limburg D, Graneto MJ, Springer J, Hamper JR, Liao S, Pawlitz JL, Kurumbail RG, Maziasz T, Talley JJ (2010). The novel benzopyran class of selective cyclooxygenase-2 inhibitors. Part 2: the second clinical candidate having a shorter and favorable human half-life. Bioorg Med Chem Lett.

[27] Berger M, Roller A, Maulide N (2017). Synthesis and antimicrobial evaluation of novel analogues of dehydroabietic acid prepared by CH-Activation. Eur J Med Chem.

[28] Abdelazeem AH, Abdelatef SA, El-Saadi MT, Omar HA, Khan SI, McCurdy CR, El-Moghazy SM (2014). Novel pyrazolopyrimidine derivatives targeting COXs and iNOS enzymes; design, synthesis and biological evaluation as potential anti-inflammatory agents. Eur J Pharm Sci.

[29] El-Nezhawy AO, Biuomy AR, Hassan FS, Ismaiel AK, Omar HA (2013). Design, synthesis and pharmacological evaluation of omeprazole-like agents with anti-inflammatory activity. Bioorg Med Chem.

